# Efficacy of Auricular Therapy for Pain Management: A Systematic Review and Meta-Analysis

**DOI:** 10.1155/2014/934670

**Published:** 2014-07-23

**Authors:** Chao Hsing Yeh, Yi Chien Chiang, Samuel L. Hoffman, Zhan Liang, Mary Lou Klem, Wilson W. S. Tam, Lung-Chang Chien, Lorna Kwai-Ping Suen

**Affiliations:** ^1^School of Nursing, University of Pittsburgh, 3500 Victoria Street, 440 Victoria Building, Pittsburgh, PA 15261, USA; ^2^Department of Nursing, Chang Gung University of Science and Technology, No. 261,Wen-hwa 1st Road, Kwei-shan, Taoyuan 333, Taiwan; ^3^Falk Library, University of Pittsburgh, 200 Scaife Hall, 3550 Terrace Street, Pittsburgh, PA 15261, USA; ^4^The Jockey Club School of Public Health and Primary Care, The Chinese Hong Kong University, Hong Kong; ^5^Division of Biostatistics, University of Texas School of Public Health San Antonio Regional Campus, Research to Advance Community Health Center, University of Texas Health Science Center at San Antonio Regional Campus, 7411 John Smith Drive, Suite 1050 Room 505, San Antonio, TX 78229, USA; ^6^School of Nursing, Hong Kong Polytechnic University, Hung Hom, Kowloon, Hong Kong

## Abstract

*Objective.* The objective of this systematic review and meta-analysis was to assess the efficacy of auricular therapy by including a sham therapy control group. *Methods.* Relevant, randomized clinical trials (RCTs) were identified by searching medical related databases from, depending on journal, 1900 (at the earliest) to 1994 (at the latest) through May 2013. The outcome measure was a pain intensity score. *Results.* Twenty-two RCTs were identified and 13 RCTs were included for meta-analysis. In these studies, auricular therapy provided significant pain relief when compared to a sham or control group. The overall standardized mean differences (SMD) was 1.59 (95% CI [−2.36, −0.82]) (13 trials, total subject numbers = 806), indicating that, on average, the mean decrease in pain score for auricular therapy group was 1.59 standard deviations greater than the mean decrease for the sham control. In terms of the efficacy of the different treatment methods, auricular acupressure boasts the largest strength of evidence for pain relief, followed by auricular acupuncture. Electroacupuncture stimulation did not show significant evidence for efficacy, which may be due to the small sample size (i.e., only 19 subjects were included). *Conclusion.* Further large-scale RCTs are needed to determine the efficacy of auricular therapy for pain.

## 1. Introduction

Pain is a highly prevalent and costly health problem in the United States. Back pain, in particular, affects at least 84% of individuals at some point during their lives [1, 2], and pain recurs in up to 80% of cases within 1 year [2]. The pain can occur at any age but is most prevalent during the third decade of life [2]. In the United States, back pain is the second most common cause of disability [3], the second leading cause of lost workplace productivity (after the common cold) [4], and the third most common reason for visiting a health provider [5]. These effects place an enormous burden on U.S. society and health care systems, as reflected by an estimated cost ranging from $84.1 billion (direct cost of health care) to $624.8 billion (indirect cost including loss of productivity) per year [6–8]. Pain in its various manifestations is also responsible for work absences, which create an enormous economic burden on individuals, families, communities, industry, and government [1, 9].

Analgesic pharmaceutical use is one of most common strategies for managing pain but it is associated with a variety of adverse side effects (e.g., drowsiness, constipation, dry mouth, gastrointestinal bleeding, and potential for addiction) [10, 11]. Pharmaceutical options are currently the first and best choice for acute pain. However, patients with chronic or recurrent pain often develop tolerance to narcotics over time and receive diminishing relief of their pain [12]. The high prevalence of extended and chronic pain highlights the need for better pain management strategies.

Complementary and alternative medicine (CAM) therapies, especially acupuncture, offer additional options in pain management [13, 14]. These CAM options tend to be cheaper, less invasive, and of lower risk than the second and third line conventional treatments of strong narcotics and invasive surgical procedures. Acupuncture can reduce the severity of pain, allowing for reduced doses of medications [14]. In a 2007 government survey, Americans had spent $33.9 billion out of pocket on CAM over the previous 12 months and an estimated $11.9 billion on visits to CAM practitioners, including acupuncturists [15, 16]. However, acupuncture currently is not covered by the majority of U.S. health care plans.

Auricular therapy is one form of acupuncture and a well-recognized element of traditional Chinese medicine (TCM) [17]. Auricular therapy is based on long-standing tradition and was modified and updated by Dr. Paul Nogier, the “father of auriculotherapy,” in the 1950s. The World Health Organization considers auricular therapy a form of microacupuncture that can affect the whole body [18]. Auricular therapy involves the relationships among the ear, energy lines (channels and meridians), and muscle regions comprising the whole body, according to a theory known as somatic reflexology. This theory posits that when a symptom or disease arises in the body, it is projected onto the ear at a regular and measureable zone [17, 19]. The TCM model views disease as being caused by the imbalance of a person's energy or* qi* [17]. The stimulation of auricular acupoints is, thus, intended to regulate* qi*, activate the meridians and collateral systems, and balance the* qi* aspects of yin and yang and, in so doing, has been successful in treating a variety of health problems, including pain [17].

Types of auricular therapy include auricular acupuncture (AA), electroacupuncture stimulation (EAS), and acupressure (AP). The former two approaches include needle insertion or application of intense electrical stimulation to ear acupoints [42]. In contrast, without needles, acupressure does not usually result in strong or painful sensations. Auricular therapy is also different from traditional body acupuncture in that auricular therapy allows needles (for AA) or acupressure patches (for AP) to remain in place up to 1 month, depending on the subject's ear and skin condition and potentially extending the therapeutic period without constant and direct provider oversight. Thus, auricular therapy can reduce both the need for patients to travel to the acupuncture site and the cost of visiting a practitioner.

Studies using auricular therapy (including AA, EAS, and AP) have shown promising effects in the pain management of several conditions, including dysmenorrhea [43–45], postoperative pain [46–48], hip fracture [22], low back pain [49, 50], and bone marrow aspiration [51]. A recent meta-analysis (including studies up to December, 2008) of auricular therapy for pain management comprising 17 studies, including three conducted in the U.S. [23, 26, 27], found that auricular therapy reduces analgesic use for perioperative pain (standard mean difference [SMD] = 0.54 (95% confidence interval (CI) 0.30, 0.77)) and reduces pain intensity for acute and chronic pain (SMD = 1.56 (95% (CI): 0.85, 2.26)) compared with control groups [52].

Studies in auricular therapy for pain management have increased since the 1980s. In order to gather and evaluate up-to-date evidence of auricular therapy efficacy for pain management, we conducted this meta-analysis, based on previous studies of systematic reviews [53] and meta-analyses [52], and expanded it to include the most current studies (up to May 30, 2013). Moreover, we included Chinese research literature in this meta-analysis because auricular therapy not only has been popular in Chinese-culture for more than 2,000 years, but also is a ubiquitous treatment for pain throughout Asia.

## 2. Methods

### 2.1. Data Sources and Searches

The literature search was performed using Ovid MEDLINE (1966 to May 2013), Ovid CINAHL (1982 to May 2013), Wiley Cochrane CENTRAL (1948 to May 2013), Embase.com (1980 to May 2013), Ovid AMED (1985 to May 2005), Ovid MANTIS (1900 to May 2013), ISI Web of Science (to May 2013), China Biological Medicine Database (CBM disc 1980 to May 2013), Chinese Medical Current Contents (CMCC) (1994 to May 2013), and China Academic Journals (CAJ) Full Text Databases (1994 to May 2013). The search keywords included* auriculotherapy*,* auricular acupuncture*,* auricular electroacupuncture*/*TENS*,* auricular acupressure*, and* laser auriculotherapy* (see example search in Table 2). For most database searches, topic search terms were combined with sensitive methodology filters designed to identify RCTs. Additional studies were identified through the references list in a recent article [52] reviewing studies of auricular therapy used for pain management. EndNote software was used to manage citations obtained through the database search.

### 2.2. Inclusion and Exclusion Criteria

In order to determine if the studies were eligible to be included in the study, two reviewers (Yi Chien Chiang and Lorna Kwai-Ping Suen) independently appraised the titles and abstracts of the English and Chinese articles. Relevant studies were retrieved, and the full articles were assessed by two independent reviewers for inclusion (Yi Chien Chiang and Lorna Kwai-Ping Suen). Any disagreement on inclusion was resolved through discussion. To be included in the analyses, the trials had to meet the following criteria: they (1) were RCTs, (2) were published in English or Chinese peer-reviewed journals, (3) compared auriculotherapy to sham and/or standard medical care with wait-list control, and (4) used a validated pain outcome measurement, including Visual Analog Scale for Pain (VAS Pain), Numeric Rating Scale for Pain (NRS Pain), or McGill Pain Questionnaire. Studies were excluded if they (1) were not RCTs, (2) combined auriculotherapy with other treatment (leading to a lack of clear evidence for efficacy), or (3) had no pain outcome measure. Recorded data included study characteristics, patient characteristics, inclusion and exclusion criteria, mode of treatment and control procedures, and outcomes. If more than one outcome measure was reported, separate evaluations were made for least and most favorable outcomes. Letters were sent to authors requesting information if we were not able to retrieve the data for meta-analysis from the article.

### 2.3. Data Synthesis and Analysis

The studies we decided to include for analysis were assessed by methodological quality (MQ) [54], which was designed for the criteria-based meta-analysis of acupuncture studies and has been accepted for use in many systematic analyses and meta-analysis of complementary therapies [55–57]. The criteria for MQ include four main categories: (1) comparability of prognosis (including method of randomization, sample size, and coverage of withdrawal and dropouts) (35 points), (2) adequate intervention (including intervention procedure, control group, and quality of the intervention) (25 points), (3) adequate effect measurement (including blinding, follow-up, remarks on side effects, and confounding variables) (30 points), and (4) data presentation and analysis (10 points). The maximum total score is 100 points; a score over 50 indicates a research report of good quality [54]. See Table 3 for detailed information.

In order to be included in the final meta-analysis, mean and standard deviation data from each study had to be retrieved. If the data were not available from the published manuscript, the authors were contacted to hopefully provide the data. Eight trials were excluded in the meta-analysis due to incomplete data. All pain intensity scores were continuous. Thus, standardized mean differences (SMD) and 95% confidence intervals (CI) were calculated to compare the pain scores between the treatment and the sham/control group in each study. A magnitude effect size (SMD) of 0.2, 0.5, and 0.8 was defined as* small*,* medium*, and* large*, respectively [58]. Random-effects models were used to estimate the combined effect, and *χ* ^2^ statistics were used to assess the heterogeneity. Additionally, *I* ^2^ statistics were also computed to show the percentage of variation due to heterogeneity [59]. Finally, publication bias was assessed by funnel plots.

## 3. Results

### 3.1. Quality Assessment

**Figure 1:**
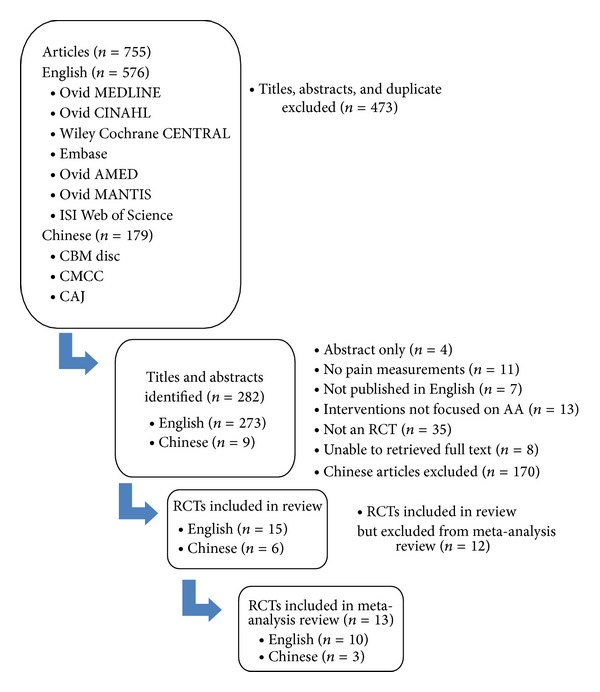
Flow chart of screened, excluded, and analyzed articles.

Figure 1 displays a flow chart of the screened, excluded, and analyzed articles that were included in the final analysis. In the English-language literature search, 273 titles and abstracts were identified, and 25 full articles were retrieved for further review. Of the 25 studies, three did not include pain outcome assessment [60–62], one was a review article [52], two had no control comparison [63, 64], and four included cointerventions, including acupuncture [65, 66], mobilization [28], and Internet information [34]. Thus, only 15 English-language studies were included for further analysis. In the Chinese-language literature search, 179 titles and abstracts were identified, and nine full articles were retrieved and reviewed. Of the nine studies, one study was a review paper [67], one study did not have a sham group [68], and one included acupuncture [69]. These three studies were therefore excluded. Ultimately, we included a total of 22 (15 English and 6 Chinese) studies in our meta-analysis, which were RCTs assessing the effect(s) of auricular therapy.

### 3.2. Characteristics of Included Studies

Table 1 lists the characteristics of the studies included for analysis. Of the 22 RCTs included, seven studies had scores of over 70 on methodological quality, with a mean score of 66.28 (SD = 8.84,  range = 45.70–79.00) for English-language studies and 54.83 (SD = 9.66,  range = 40.50–70.00) for Chinese-language studies. The mean scores of English-language studies were significantly higher than Chinese-language studies (*P* = 0.024). One English-language study [26] and one Chinese language study [38] scored less than 50, which indicates a lower MS. The countries where the studies were conducted included Europe (*n* = 9), the United States (*n* = 5), China (*n* = 6), and Taiwan (*n* = 2). Studies conducted in the United States took place mainly in military settings (*n* = 3). The sample size ranged from 19 to 180, with a mean of 62. Studies conducted in China or Taiwan tended to have larger sample size (*n* ≥ 60).

A VAS scale (0–10  or  0–100) was used for all pain outcome measures. Among the 22 trials, auricular therapy methods included AA (*n* = 10), EAS (*n* = 4), and AP (*n* = 8). The type of pain included perioperative pain (*n* = 9), acute pain (*n* = 7), and chronic pain (*n* = 6). The treatment duration ranged from one treatment (AA) to weekly AA treatment for up to 6 weeks [24]. The most popular acupoints selected for treatment were* corresponding points* (*n* = 20),* shenmen* (*n* = 17), and* subcortex* (also called dermis) (*n* = 8). Seven trials included a sham control (using sham acupoints) for comparison of auricular effects in pain relief, and the selection of sham acupoints was based on using points outside the pain zone area on the ear. An electrical point finder was used in most cases to find the acupoints for treatment (*n* = 8). Bilateral acupoints were used for treatment in six trials, and unilateral acupoints were used in four trials. Twelve trials did not specify whether bilateral or unilateral acupoints were used. Most of the trials reported positive outcomes; however, one trial showed AA was less effective when compared to local analgesic use [27]; two trials reported mixed results (multiple times points of pain scores) [13, 23].

### 3.3. Meta-Analysis (Effects of Intervention)

In the final meta-analysis, nine studies were excluded because we were not able to retrieve raw data (i.e., mean and standard deviation), which included five English-language studies [22, 23, 25, 30, 70] and four Chinese-language studies [37–40]. Due to the different methods of treatment (including AA, EAS, and AP) and great variation of study endpoints among the trials, findings in this meta-analysis were presented according to different treatment methods (follow-up duration, which included immediate (within 15 minutes), 12 to 24 hours after treatment, 24 to 48 hours after treatment, and long term follow-up).

### 3.4. Overall Pain Relief of Auricular Therapy for 13 Studies

**Figure 2:**
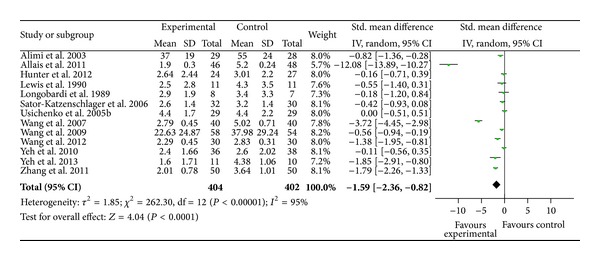
Pain relief of auricular therapy for 13 trials.

Figure 2 presents the findings of the 13 trials included for meta-analysis. Among these 13 trials, two studies [20, 43] used a 0–100 scale to measure pain, while the eleven other studies used a 0–10 scale. Seven studies reported statistically significant pain relief of auricular therapy compared to the sham group [20, 21, 32, 34, 36, 39, 41, 43], while six studies found no significant difference in pain relief between auricular therapy and the sham control [24, 26, 27, 29, 31, 32]. Among the 13 trials, auricular therapy was found to be a significant method of pain relief when compared to the sham or control group (SMD = − 1.59,  95%  CI  [−2.36, − 0.82], *P* = 0.001). Highly significant heterogeneity was found among the 13 studies (*χ* ^2^ = 262.30, *I* ^2^ = 95%, *P* < 0.01), indicating their heterogeneity. We conducted further sensitivity testing and removed two studies that showed much larger effect than the other studies [21, 35]. In doing this, heterogeneity was reduced (*χ* ^2^ = 51.23,  *I* ^2^ = 80%,  *P* < 0.01) and the SMD decreased to 0.69 (95%  CI  [−1.08, − 0.30]). The overall strength of the evidence for the efficacy of auricular therapy for pain relief was rated as* medium* to* large*.

### 3.5. Pain Relief vis-à-vis Different Auricular Therapy Treatment Methods

**Figure 3:**
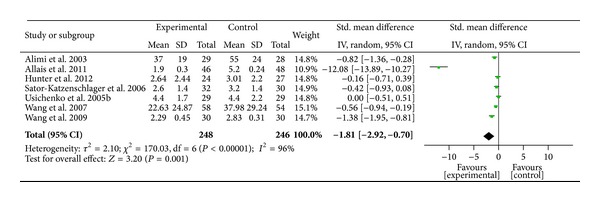
Pain relief of auricular therapy using auricular acupuncture.

**Figure 4:**
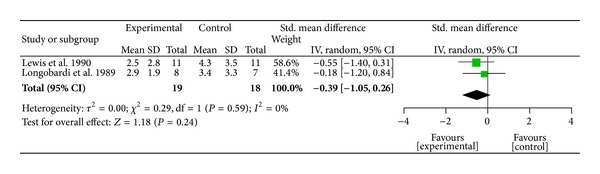
Pain relief of auricular therapy using electroacupuncture stimulation.

**Figure 5:**
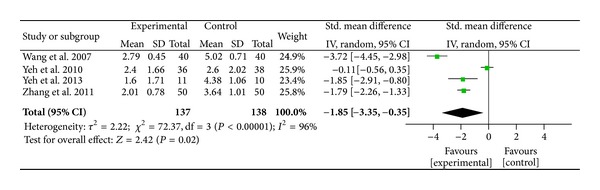
Pain relief of auricular therapy using auricular acupressure.

Among the seven studies featuring AA, AA was found to be a significant method of pain relief when compared to the sham or control group (SMD = − 1.81,  95%  CI  [−2.92, − 0.70], *P* = 0.001) (Figure 3). Highly significant heterogeneity was found among the studies (*χ* ^2^ = 170.03, *I* ^2^ = 96%,  *P* < 0.001), indicating their heterogeneity. Publication bias was assessed by funnel plot and asymmetry was observed, which suggested potential publication bias due to the study by Allais et al. [21]. We conducted further sensitivity testing and removed the Allais et al. study. After removal of the study [21], AA was found to be significant for pain relief when compared to the sham or control group (SMD = − 0.55,  95%  CI  [−0.91, −0.19],  *P* = 0.003, *n* = 202). The overall strength of the evidence for the efficacy of auricular therapy for pain relief was rated as* medium* to* large*. Among the two studies using EAS, EAS was found to be nonsignificant for pain reduction when compared to the sham or control group (SMD = − 0.39;  95%  CI  [−1.05, 0.26]; *P* = 0.24, *n* = 19) (Figure 4). Among the four studies using AP, AP was found to be a significant method for pain relief when compared to the sham or control group (SMD = −1.85,95%  CI  [−3.35, − 0.35],  *P* = 0.002) (Figure 5). The overall strength of the evidence for the efficacy of auricular therapy for pain relief was rated as* large*.

### 3.6. Immediate Pain Relief after Auricular Therapy (within 15 Minutes after Treatment)

**Figure 6:**
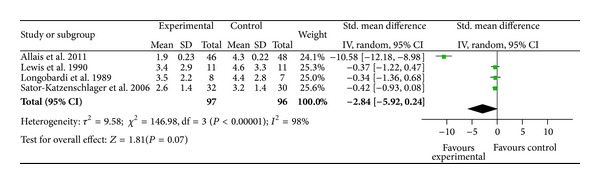
Immediate pain relief after auricular therapy (within 15 minutes).

Four studies compared the immediate pain relief of auricular therapy (15 minutes or less) for treating migraine using AA [21], pain with burns using EAS [26], perioperative pain during oocyte aspiration in IVF treatment using EAS [29], and distal extremity pain using EAS [27]. Heterogeneity tests were significant (*χ* ^2^ = 146.98, *I* ^2^ = 98%, *P* < 0.01), which indicates statistical evidence for differences between the four studies (Figure 6). Intervention groups tended to have lower scores of pain intensity than sham groups; however, only one study reached statistical significance [21]. The combined mean difference for the four studies showed nonsignificant pain reduction for immediate effect measures (SMD = − 2.84; 95%  CI  [−5.92, 0.24]; *P* = 0.07,  *n* = 193).

### 3.7. Pain Relief after Auricular Therapy (12 to 24 Hours after Treatment)

**Figure 7:**
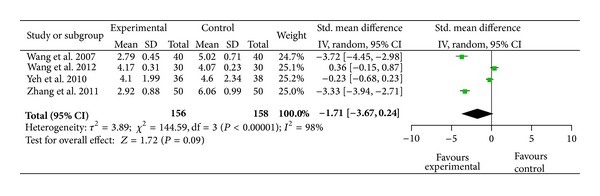
Immediate pain relief after auricular therapy (12 to 24 hours after treatment).

**Figure 8:**
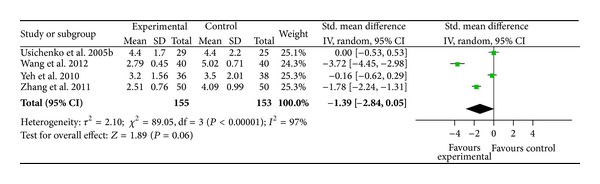
Pain relief after auricular therapy (24 to 48 hours after treatment).

Four studies included pain intensity measured at 12 to 24 hours after auricular therapy. The four studies showed significant heterogeneity (*χ* ^2^ = 144.59, *P* < 0.001) (Figure 7). Two studies had significant pain relief at 12 to 24 hours after auricular therapy [36, 39]. The combined mean difference for these four studies did not reach statistical significance (SMD = − 1.71;  95%  CI  [−3.67, 0.24]; *P* = 0.09; *n* = 314) (Figure 8).

### 3.8. Pain Relief after Auricular Therapy (24 to 48 Hours after Treatment)

Four studies examined pain relief at 24 to 48 hours after auricular therapy and displayed good quality [31, 32, 39, 41]. Heterogeneity between studies was highly significant (*χ* ^2^ = 89.05, *P* < 0.001) (Figure 8). Two studies demonstrated statistically significant pain relief after auricular therapy at 24 to 48 hours [39, 41], while the other two trials did not show pain relief [31, 39]. Among the four trials, auricular therapy did not show significant pain relief after 24 to 48 hours when compared to sham groups (SMD = − 1.39;  95%  CI  [−2.84, 0.05]; *P* = 0.006; *n* = 306).

## 4. Discussion

In this meta-analysis, we had the advantage of including both English- and Chinese-language studies, which was not done in Asher et al.'s previous meta-analysis [52]. The main finding of this study is that auricular therapy provided significant pain relief when compared to sham or control groups in the various studies analyzed. The overall SMD was 1.59 (95%  CI  [−2.36, − 0.82]) (13 trials, total subject number = 806), indicating that, on average, the mean decrease in pain score for auricular therapy group was 1.59 standard deviations greater than the mean decrease for the sham control, which is similar to the earlier meta-analysis (SMD = 1.56, 95%  CI  [0.82, 2.26]) (8 trials, *n* = 387) [52]. Findings from that meta-analysis and our meta-analysis demonstrate significant heterogeneity of the studies included. When attempts were made to remove particular individual studies, heterogeneity was reduced but still existed, and SMD dropped to −0.69 (*I* ^2^ = 80%,  CI:   − 1.08, − 0.30) for this study and 1.01 (*I* ^2^ = 74%, 95%   CI:  0.51, 1.51) for Asher et al.'s study [52]. Compared to Asher et al., which included six studies (*n* = 303) in the meta-analysis, our meta-analysis had 13 studies with larger sample size (*n* = 806), which leads to a smaller effect size. Hence, our study more accurately represents the efficacy of auricular therapy for pain relief, with moderate- to high-strength evidence.

Before interpreting the findings of this study, we need to be aware of its limitations. First, the results of this meta-analysis depend on the quality of the studies included. The quality of the studies included had MQ mean scores of 65.82 for English-language studies and 54.83 for Chinese-language studies. According to the definition of MQ, a score of 50 indicates* good* quality. In the studies included in this meta-analysis, 91% were rated as* good*, which is much higher than the previous meta-analysis which had only 35% rated as* good* [52]. Moreover, the differences in study parameters are likely responsible for the varying treatment effect due to significant variation, including acupoint selection, form of auricular therapy, treatment duration, and study endpoints—all of which could impact the quality of the studies. Second, we only included published studies, which may skew the results because most studies with negative results were unpublished. Finally, only seven studies (32%) used a sham group for comparison. Without the sham comparison, the effect of auricular therapy for pain is subject to placebo effects, patient expectations on treatment outcome, and given patient's relationship with his or her therapist.

Acupoint selection for both treatment and sham control groups is a critical factor impacting treatment effects. Differing from body acupuncture, which works on the meridian basis (i.e., health is promoted through the balancing of* yin* and* yang*), auricular therapy works on a microsystem basis, with the ear as a self-contained microsystem that can affect the state of the entire body. Based on Nogier's theory [71], somatotopic correspondences can be found between auricular points and their corresponding (i.e., projected) body areas. When a person suffers from disease in a particular part of the body, the auricular acupoints may show not only a decrease in auricular cutaneous electrical resistance, but also a decrease in pain threshold [72]. Thus,* corresponding acupoints* are the key acupoints for treatment in auricular therapy. In this meta-analysis,* corresponding acupoints* (91%, *n* = 20) and* shenmen* (a general analgesic point) (77%, *n* = 17) are the most commonly used acupoints for pain treatment. The positive outcomes for the majority of studies included in this meta-analysis suggest that auricular acupoints are indeed specific to particular diseases or symptoms. However, we need to be cautious about this conclusion because only 32% (*n* = 7) of the total studies included in our meta-analysis had a sham group comparison.

In the studies that included a sham group comparison, the selection of sham acupoints consisted of simply placing them outside the corresponding pain area, which is reasonable considering the somatotopic mapping between auricular acupoints and body. However, none of these studies acknowledged that the selection of these sham acupoints may conflict with TCM meridian and channel theory. In other words, what is intended to be a sham acupoint in terms of one auricular paradigm could actually influence an intervention's outcomes, positively or negatively, according to another auricular paradigm.

Auricular therapy, a form of acupuncture, has a history of more than 2,000 years in TCM. Therefore, it still shares some key treatment parameters of TCM meridian and channel theory that were followed by the researchers of the studies assessed in this meta-analysis. For example, some auricular acupoints are one-point, multiple-disease, according to TCM* zang-fun* theory. In one study, the* lung* acupoint was used to reduce pain during incisions [27], based on the association between* lung* and the body skin surface in TCM theory. However, to date, there have been no empirical studies that discuss the treatment outcomes of acupoint selection by comparing the projection of somatic topography with TCM meridian and channel theory. In addition, although auricular acupoints are basically the same between the Chinese and Nogier systems, the ear charts do vary in somatotopic arrangement, particularly with regard to spinal, internal organ, and reproductive organ location [17]. Therefore, more empirical evidence is needed to establish the validity of ear charts between the Chinese and Nogier approaches to auricular therapy.

Treatment duration (i.e., how long the treatments provide benefit) is another key factor for treatment outcomes—particularly for chronic pain. In this meta-analysis, the treatment duration for chronic pain varied from one-time treatment to up to six treatments. The lack of agreement on the optimal duration of auricular therapy may mean that some patients do not receive the best treatment. In general, 2 to 10 weeks of auricular therapy has been reported to provide treatment benefits [17, 19]; however, these reports lack empirical evidence. In acupuncture, six treatments over a 3-week period provide better pain relief than does the administration of fewer than six treatments [73]. Studies on the sustained effects of auricular therapy for pain relief are varied and report findings ranging from immediately [21] to up to 6 months [24], but the findings are limited by small sample size [52]. In a study of AP for chronic low back pain, 1 week of AP can reduce pain intensity by 45% [74], and 4 weeks of APA can achieve even more pain relief (i.e., 75% reduction) and maintain such effects for 1 month [33]. Studies have established evidence of auricular therapy, yet more empirical studies are needed to establish an adequate assessment of treatment duration.

In terms of the efficacy of the different treatment methods, AP boasts the largest strength of evidence for the efficacy for pain relief, followed by AA. EAS did not show significant evidence for efficacy, which may be due to the small sample size (i.e., only 19 subjects were included). Compared to AP, AA and EAS are more popular in Europe and the United States. Additionally, these techniques usually need to be performed by qualified physicians. In contrast, studies conducted in China and Taiwan primarily feature AP, and AP is a noninvasive, low-cost, and self-managed approach for patients. The application of AP utilizes botanical plant seeds or magnetic pellets taped on the both sides of the ears to stimulate acupoints. Once seeds are applied with tape by a trained therapist, the taped-on seeds can remain on the ears for 1 to 3 weeks, depending on the skin condition of the ear. The biggest advantage of AP is that patients themselves can stimulate the acupoints by pressing them with the thumb and forefinger as directed to achieve acupuncture-like effects. Patients are instructed to press the acupoints whenever they want to decrease pain. With this patient involvement, fewer visits to a therapist are required for auricular acupressure compared to body acupuncture. Although auricular therapy (especially AP) can be easily administered by a trained practitioner, further research should investigate whether or not it could be self-administered and whether or not accurate seed placement could be achieved by the patient.

A definitive elucidation of the underlying biological mechanism of auricular therapy in treating pain remains elusive. One theoretical explanation of auricular therapy is that pain and neuronal excitability are relieved by normalizing pathological, hypersensitive reflex pathways (i.e., the neural immune pathway) that interconnect the ear microsystem and the somatotopic region of the brain [17, 19]. The neurophysiological connections between ear acupoints and the human CNS have been corroborated by fMRI [75]. The stimulation of acupoints is thought to cause vasodilative effects by releasing either beta-endorphin to elicit short-term analgesic effects or neuropeptide-induced anti-inflammatory cytokines for long-term effects [76–78]. Considering the complex interaction between cytokines, neuropeptides, and neurotrophins pertaining to chronic pain, possible pathways of the ameliorating effect of auricular therapy on pain include (1) the downregulation of proinflammatory cytokines and the upregulation of anti-inflammatory cytokines, (2) the downregulation of proinflammatory neuropeptides (e.g., calcitonin gene-related peptide), and (3) the downregulation of neurotrophins (e.g., nerve growth factor, NGF) [77, 79]. These responses may be modulated by inflammatory mediators and could explain the analgesic effects of auricular therapy.

## 5. Conclusion

Our findings suggest that auricular therapy can be used as an adjunct therapy for pain management and, therefore, reduce analgesic use to minimize potential adverse effects and tolerance. Nonetheless, further studies—particularly large scale of RCTs—are needed to further confirm the efficacy of auricular therapy for pain and must take into consideration important features of methodological design, which include point specification, stimulation, treatment duration, placebo effects, and patient expectations of treatment outcomes.

## Figures and Tables

**Table 1:** Studies of auricular therapy for pain management.

Author (year of publication)	Sample			Power estimate	Blinding			Intention to treat	Diagnosis	Side effects	Site of study	Acupuncture point selection		Method to locate points	Treatment session	Side treated
	Size	Intervention	Control		Acupuncture	Patient	Data collector					Point selection	Sham selection			
Alimi et al. (2003) [20]	90	AA [*n* = 29]	Sham AA [*n* = 31] Sham AP [*n* = 30]	0.9, *α*: 0.05	No	Yes	Yes	Yes (no dropout)	Cancer pain	None	France	Electrodermal response at the points on the ear where pain projected	Outside corresponding pain area	Electronic	2 tx, 1 m apart	Not specified

Allais et al. (2011) [21]	89	AA [*n* = 43]	Sham [*n* = 46]	n/a	No	Yes	Yes	No	Migraine attack	Unbearable exacerbation of pain (*n* = 4)	Italy	Head zone (using pain pressure test)	Sciatic nerve zone	Alogometric	1 tx (no mention of duration)	Not specified

Barker et al. (2006) [22]	38	AP [*n* = 18]	Sham AP [*n* = 20]	0.8, *α*: 0.05	Yes	Yes	Yes	Yes (no dropout)	Hip fracture	n/a	Austria	Shenmen, hip, valium point	Tip of the concha	None specified	1 tx	Bilateral

Goertz et al. (2006) [23]	100	AA [*n* = 50]	Standard care [*n* = 50]	n/a	No	No	Yes	Yes	Acute pain	n/a	U.S.	Cingulate gyrus and thalamic nuclei (mediate acupuncture analgesia)	None	None specified	1 tx (4–6 days until fell out)	Not specified

Hunter et al. (2012) [24]	51	AA + exercise [*n* = 24]	Exercise alone [*n* = 27]	0.8, *α*: 0.05, (56/group in full trial)	No	No	Yes	No	Chronic low back pain	Pain, redness, minor bleeding	North Ireland	Shenmen, lumbar spine and cushion	n/a	None specified	6 tx (AA for 48 hrs until fell out), 6 wks	Not specified

Kindberg et al. (2009) [25]	207	AA [*n* = 105]	Local anesthetics [*n* = 102]	0.8, *α*: 0.05	No	No	Yes	Yes	Intended vaginal delivery	None	Denmark	Helix (genital), shenmen, bladder 36, governor vessel 20 (general relaxation) (4 points)	n/a	None specified	1 tx	Bilateral

Lewis et al. (1990) [26]	11	EA [*n* = 11]	Placebo pill [*n* = 11] (crossover)	n/a	No	Yes	No	No	Burns with pain	n/a	U.S.	Shenmen, lung, dermis, three more per burn locale (6 points)	Placebo pill	Electronic	1 tx	Bilateral

Longobardi et al. (1989) [27]	15	EA [*n* = 8]	Sham [*n* = 7]	n/a	No	Yes	No	No	Distal extremity pain	n/a	U.S.	Shenmen, lung, dermis, two more due to painful extremity (corresponding points)	n/a	Electronic	1 tx (10 min)	Bilateral

Romoli et al. (2000) [28]	60	EAS + mobilization [*n* = 20]	Body EAS + mobilization [*n* = 20], mobilization [*n* = 20]	0.9, *α*: 0.05	No	No	Yes	No	Shoulder pain (chronic pain)	n/a	Italy	Corresponding point of shoulder	n/a	Probe	1 tx (15–20 min), 5 wks	Bilateral

Sator- Katzenschlager et al. (2006) [29]	94	AA [*n* = 32] EAS [*n* = 32]	Control [*n* = 30]	0.8, *α*: 0.05	n/a	Yes	Yes	Yes	Perioperative pain	None	Austria	Uterus, shenmen and cushion	n/a	Electronic	1 tx (3 hrs)	Dominant side

Usichenko et al. (2005) [30]	18	AA [*n* = 10]	Sham [*n* = 8]	n/a	No	Yes	Yes	No	Post-knee surgery pain	None	Germany	Shenmen, lung, knee	Nonacupuncture points (helix)	Electronic	1 tx	Not specified

Usichenko et al. (2005) [31]	54	AA [*n* = 29]	Sham [*n* = 25]	0.8, *α*: 0.05	Yes	Yes	Yes	No	Post-hip surgery pain	Pain from needle, hemorrhages, headache	Germany	Shenmen, lung, thalamus and hip points	Nonacupuncture points (helix)	Electronic	1 tx (stay for 3 days)	Not specified

Yeh et al. (2010) [32]	74	AP [*n* = 36]	Control [*n* = 38]	0.8, *α*: 0.05	No	Yes	No	No	Postoperative pain	n/a	Taiwan	Shenmen, occipital, lumbar-sacrum vertebra, stomach, cardia, endocrine	n/a	Identified by two clinical acupuncture experts	1 tx (72 hrs)	Unilateral

Yeh et al. (2013) [33]	19	AP [*n* = 10]	Sham [*n* = 9]	n/a	No	Yes	No	No	Chronic low back pain	Allergy to tape, pain on ear points	U.S.	Shenmen, subcortex, sympathetic, corresponding points	Stomach, duodenum, mouth, kidney	Electronic	Weekly tx, 4 wks	Bilateral

Yeh et al. (2013) [34]	100	AP + interactive Internet based intervention [*n* = 50]	AP only [*n* = 50]	0.8, *α*: 0.05	n/a	n/a	n/a	No	Dysmenorrhea	n/a	Taiwan	Shenmen, kidney, liver, internal genitals, central rim, endocrine	n/a	Identified by two acupuncturists	1 tx (after pain relief 48 hrs later)	Not specified

Wang et al. (2009) [35]	159	AA [*n* = 58]	Sham [*n* = 54]; Control [*n* = 47]	0.85, *α*: 0.05	No	Yes	Yes	No	Low back and/or posterior pelvic pain	n/a	U.S.	Shenmen, kidney, analgesia	Wrist, shoulder, extra auricular points	Zone system	1 tx (1 wk)	Unilateral

Wang et al. (2007) [36]	80	AP [*n* = 40]	Usual care [*n* = 40]	n/a	n/a	n/a	n/a	No	Laparoscopic cholecystotomy	n/a	China	Shenmen, subcortex, sympathetic, pancreas, bile, liver, shoulder, endocrine, duodenum, spleen	n/a	None specified	1 tx (press 20 min/ time, 3 times/day)	Not specified

Yang and Zhang (2011) [37]	60	AP [*n* = 30]	Pain medication [*n* = 30]	n/a	n/a	n/a	n/a	No	Hemorrhoid	n/a	China	Shenmen, subcortex, corresponding point (anal)	n/a	Probe	1 tx (press 30–50 times, 3–4 times/day)	Unilateral

Ma and Fang (2011) [38]	80	AP [*n* = 40]	Usual care and pain medication [*n* = 40]	n/a	n/a	n/a	n/a	No	Renal colic pain	n/a	China	Kidney, sympathetic, ureter, *san jiao *	n/a	None specified	1 tx (press 1-2 min, repeat per 5 minutes)	Not specified

Zhang and Tzu (2011) [39]	100	AP [*n* = 50]	Usual care [*n* = 50]	n/a	n/a	n/a	n/a	No	Replantation of digits	n/a	China	Shenmen, subcortex, sympathetic, corresponding point	n/a	None specified	1 tx (press 3–5 min, 3 times/day)	Bilateral

Li et al. (2012) [40]	180	EAS [*n* = 60]	EAS [*n* = 60], Control [*n* = 60]	n/a	n/a	n/a	n/a	No	Cesarean section	n/a	China	Shenmen	Eye	None specified	1 tx (stim. 30 min, repeat at 4th, 10th, 22nd hr)	Not specified

Wang et al. (2012) [41]	60	AA [*n* = 30]	No AA [*n* = 30]	n/a	n/a	n/a	n/a	No	Hip replacement	n/a	China	Shenmen, kidney, subcortex hip	n/a	None specified	1 tx (press 5–10 min/time, 4 times/day)	Not specified

AA: auricular acupuncture; EAS: auricular electroacupuncture stimulation/TENS; AP: auricular acupressure; n/a: no report.

**Table 2:** Ovid MEDLINE search for auricular therapy for pain management article.

Search string	
(1) Acupuncture	
(2) Acupuncture, ear	
(3) Auriculotherapy	
(4) Ear, external	
(5) Acupuncture therapy or acupressure	
(6) 4 and 5	
(7) Auriculotherapy.ti,ab.	
(8) Auricular therapy.ti,ab.	
(9) Aural therapy.ti,ab.	
(10) Ear acupuncture.ti,ab.	
(11) Ear acupressure.ti,ab.	
(12) Auricular acupuncture.ti,ab.	
(13) Auricular acupressure.ti,ab.	
(14) 1 or 2 or 5 or 6 or 7 or 8 or 9 or 10 or 11 or 12	
(15) Limit 13 to “therapy (sensitivity)”	

**Table 3:** Methodological quality (developed by Ter Riet) (score 0–100).

Study population (35 points)	
(A) Homogeneity (2)	
(B) Comparability of prognoses at baseline (5)	
(C) Adequate randomization procedure (4)	
(D) Dropouts described for each treatment group separately (4)	
(E) Loss to follow-up (8)	
(F) Study size (12)	
Intervention (25 points)	
(G) Interventions included in protocol and described (10)	
(H) Pragmatic study (5)	
(I) Cointerventions avoided (or comparable) (5)	
(J) Placebo-controlled (5)	
Measurement of effect (30 points)	
(K) Patient blinded (5)	
(L) Outcome measures relevant (10)	
(M) Validity/reliability of instruments (5)	
(N) Blinded outcomes assessments (5)	
(O) Follow-up period adequate (5)	
Data presentation and analysis (10 points)	
(P) Intention-to-treat analysis (5)	
(Q) Data presented for most important outcome measures (5)	
